# Progress in Traditional Chinese Medicine Against Respiratory Viruses: A Review

**DOI:** 10.3389/fphar.2021.743623

**Published:** 2021-08-31

**Authors:** Bao-Hong Li, Zhong-Yuan Li, Miao-Miao Liu, Jing-Zhen Tian, Qing-Hua Cui

**Affiliations:** ^1^ College of Pharmacy, Shandong University of Traditional Chinese Medicine, Jinan, China; ^2^ Innovation Research Institute of Traditional Chinese Medicine, Shandong University of Traditional Chinese Medicine, Jinan, China; ^3^ Qingdao Academy of Chinese Medicinal Sciences, Shandong University of Traditional Chinese Medicine, Qingdao, China

**Keywords:** traditional Chinese medicine, respiratory virus, basic theory of traditional Chinese medicine, clearing heat and detoxication, mechanism of action, COVID-19

## Abstract

Respiratory viruses, such as severe acute respiratory syndrome coronavirus (SARS-CoV)-1, SARS-CoV-2, influenza A viruses, and respiratory syncytial virus, pose a serious threat to society. Based on the guiding principles of “holism” and “syndrome differentiation and treatment”, traditional Chinese medicine (TCM) has unique advantages in the treatment of respiratory virus diseases owing to the synergistic effect of multiple components and targets, which prevents drug resistance from arising. According to TCM theory, there are two main strategies in antiviral treatments, namely “dispelling evil” and “fu zheng”. Dispelling evil corresponds to the direct inhibition of virus growth and fu zheng corresponds to immune regulation, inflammation control, and tissue protection in the host. In this review, current progress in using TCMs against respiratory viruses is summarized according to modern biological theories. The prospects for developing TCMs against respiratory viruses is discussed to provide a reference for the research and development of innovative TCMs with multiple components, multiple targets, and low toxicity.

## Introduction

Viral diseases that pose a serious threat to society occur frequently, and preventing and treating viral infections have become major scientific problems. In particular, respiratory viruses have high infectivity and high incidence. Virus variability, drug resistance, and the high risks of drug research and development have resulted in there being only a handful of drugs for treating viral diseases.

Chinese herbs are the pharmaceutical ingredients that are collected, processed, and prepared according to the basic theory of traditional Chinese medicine (TCM), which explains the mechanism of action and guides clinical applications. Most TCMs are plant-based; thus, there is a saying that “all kinds of herbs are grass-based”. The prevention and treatment of viral diseases with TCM has a long history and clinical practice, from the Treatise on Febrile Diseases written around 2000 years ago and the Treatise on Pestilence in the Ming (1,368–1,644) and Qing (1,644–1911/12) dynasties, to the prevention and control of viral diseases in the modern era, reflects the advantages of TCM in this field (Zhang et al., 2019; Zhu et al., 2021). At the end of 2019, an outbreak of a novel coronavirus, severe acute respiratory syndrome coronavirus (SARS-CoV)-2, quickly became a pandemic. Although many countries worldwide struggled to combat the spread of the virus, China rapidly controlled the outbreak, and TCM played an important role in treating coronavirus disease 2019 (COVID-19).

According to the TCM characteristics and the characteristics and pathogenic mechanism of respiratory viral diseases, this review systematically describes the relationship among virus, host, and TCMs to provide a TCM strategy for treating respiratory viruses.

### Overview of Respiratory Viruses

Morbidity and mortality due to respiratory diseases are high worldwide (Burney et al., 2015), and 90% of respiratory infections are caused by viruses, the majority of which are RNA viruses, such as orthomyxoviruses, paramyxoviruses, and coronaviruses, and rhinoviruses, and some of which are DNA viruses, such as adenoviruses. Orthomyxoviruses include influenza A virus (IAV) and influenza B virus (IBV), which are characterized by segmental RNA, variation, hemagglutination, and absence of hemolysis. Paramyxoviruses include respiratory syncytial virus (RSV), parainfluenza virus, measles virus, and mumps virus, and they have a low frequency of RNA mutation in different segments and show hemagglutination and hemolytic activity. Coronaviruses include the SARS-CoV-2, SARS-CoV, and MERS-CoV novel coronaviruses, which have high pathogenicity and variability (Battles and McLellan, 2019; Abdelrahman et al., 2020).

Respiratory viruses are highly contagious and transmitted mainly through respiratory secretions, stools, urine, droplets, air and contact (Weber and Stilianakis, 2021). Most respiratory viruses occur in seasonal outbreaks, with infants, the elderly, and immune-compromised populations at high risk (Nichols et al., 2008), and the prevalence and severity vary across geographical regions and populations (Moriyama et al., 2020). Infection often causes oral, nasal, and pharynx discomfort, airway inflammation, and lung injury, and serious cytokine storms may result in acute respiratory distress and multiple organ failure, and even lead to death of patients (Abdelrahman et al., 2020). For example, the Spanish flu, which began in 1918, killed tens of millions of people and the outbreak of the H1N1 virus in 2009 killed hundreds of thousands of people worldwide (Garten et al., 2009; Shieh et al., 2009). By June 2021, SARS-CoV-2, which was detected at the end of 2019, has infected nearly 200 million people, and killed more than 3.5 million people.

Thus, the prevention and treatment of respiratory virus diseases is a crucial global health issue. Guided by the basic theory of TCM, TCMs have unique advantages in the prevention and treatment of respiratory viruses through the overall regulation of human immune function due to its multi-component and multi-target characteristics.

### Basic Theory of TCM

The basic theory of TCM has “holism” as its guiding principle and “syndrome differentiation and treatment” as its method of diagnosis and treatment, guiding the use of TCMs against viruses. The principle of holism regards the body as an organic whole, and understands the occurrence and development of local diseases as related to the whole; thus, local diseases can only be treated effectively by considering the whole body. The concepts of syndrome differentiation and treatment are defined as follows. Syndrome differentiation is the process of proving and distinguishing the type of disease, that is, knowing the location, etiology, properties, and the relationship between the “zheng (energy)” and “xie (evil)” of the disease, which reflect the nature of pathological changes. Treatment is the process of identifying the appropriate treatment methods according to the results of syndrome differentiation. This also corresponds to the "personalized treatment" in modern medicine, which is of great significance in diagnosis and treatment (Li and Xu, 2011; Ma et al., 2019).

According to TCM theory, there are two main ways that antiviral TCMs work, which are “dispelling evil” and “fu zheng”. Dispelling evil refers to the elimination of viruses, which is usually direct inhibition or killing of viruses by Chinese herbs. The mechanism of action of this kind of herb is like that of direct-acting antiviral drugs in Western medicine (direct-acting antivirals). Fu zheng refers to improving the body’s physical fitness and ability to resist evil and to rehabilitation; zheng qi is stored in the body, and prevents evil, which is also an important aspect of the antiviral mechanisms of TCMs. These two modes of action are also reflected in the mechanism of TCM treatment of respiratory viral diseases (Wang et al., 2020a).

### Antiviral Research Guided by Holism and Syndrome Differentiation and Treatment

**FIGURE 1:**
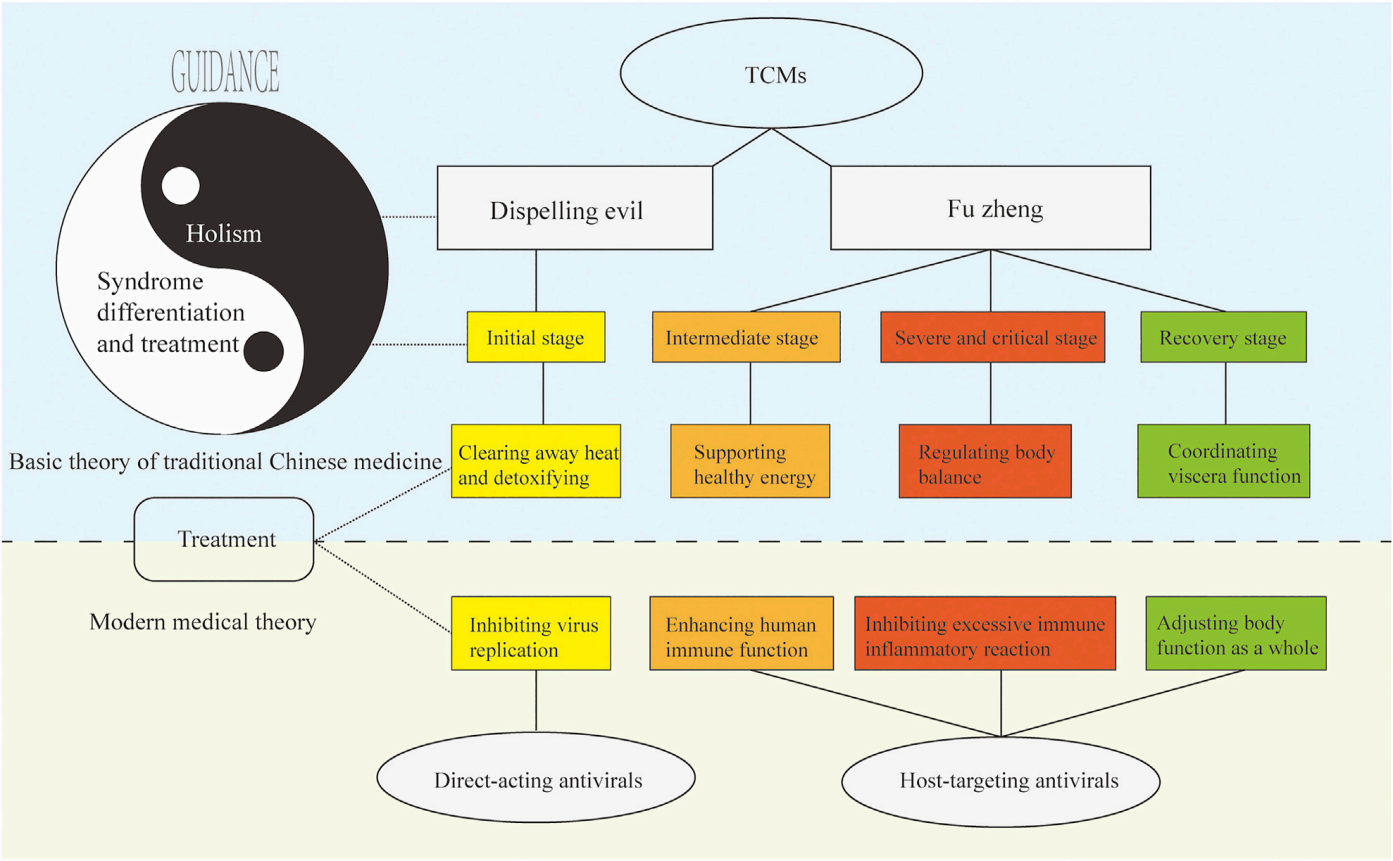
TCM antiviral theory based on the basic theory of TCM and its relationship with modern medicine. Under the guidance of “holism” and “syndrome differentiation and treatment”, TCM can remove pathogenic factors and strengthen the body, to achieve the effect of preventing and treating viral diseases, which is corresponding to the modern medical system.

Figure 1 shows the relationship between TCM antiviral theory with respect to the basic theory of TCM and modern medicine. We discuss treatment of COVID-19 with TCM as an example of using the principles of holism and syndrome differentiation and treatment. In TCM theory, COVID-19 belongs to the category of epidemic disease. COVID-19 is caused by the invasion of the exogenous pathogen SARS-CoV-2 and the deterioration in human immune function, which exacerbates the imbalance in the body (excessive immune inflammatory reaction) and causes organ dysfunction. From the perspective of holism, COVID-19 is a struggle between the virus and the immune system, which leads to an imbalance in the homeostasis of the human internal environment. From the perspective of syndrome differentiation and treatment, the progress of the disease can be divided into the initial stage, the intermediate stage, the severe or critical stage, and the recovery stage. Different treatments target different stages of the disease; for example, clearing heat and detoxification in the early stage are part of dispelling evil in TCM. The latter three stages require the suppression of an excessive immune response and inflammation, regulation of balance in the body, and coordination of the functions of the viscera, all of which are part of fu zheng in TCM. Clinical results have shown that TCM treatment is effective for COVID-19, especially in significantly reducing the number of patients transitioning from the early and middle stages of the disease to severe and critical illness, which is key to reducing the incidence and mortality of critical illness (Lee et al., 2021).

## Research Status of Antiviral TCMs

This review focuses on five Chinese herbal medicines (Isatidis radix, Glycyrrhizae radix et rhizoma, Scutellariae radix, Houttuyniae herba, and Chebulae fructus), six traditional Chinese formulae (Ge-gen decoction [GGD], San-wu-huang-qin decoction [SWHQD], Gu-ben-fang-xiao decoction [GBFXD], Qing-fei-pai-du decoction [QFPDD], Ma-xing-shi-gan decoction [MXSGD], and Ma-huang-xi-xin-fu-zi decoction [MXFD]), six proprietary Chinese medicines (Lian-hua-qing-wen capsules [LHQWC], Yin-hua-ping-gan granules [YHPGG], Shu-feng-jie-du capsules [SFJDC], Re-du-ning injection [RDNI], Xue-bi-jing injection [XBJI], and Tan-re-qing injection [TRQI]), and six active ingredients from natural products that have been studied extensively (forsythin, rheum emodin, baicalein, baicalin, quercetin, and glycyrrhizic acid) (Table 1). Based on a modern biological interpretation of TCM antiviral theory, we discuss the efficacy and mechanism of TCMs against respiratory viruses from the perspectives of the direct effect on viruses (Table 2), immune regulation (Table 3), control of inflammatory factors (Table 4), and tissue protection (Table 5).

**TABLE 1:** Overview of antiviral TCMs.

Type	TCM	Parts/components	Main active components	References
Chinese herbal medicine	*Isatis indigotica* Fort	Root	Alkaloids, organic acids, nucleosides, amino acids, flax lignans, flavonoids, sterols, volatile oils, polysaccharides etc.	Zhou and Zhang (2013)
	*Glycyrrhiza uralensis* Fisch., *Glycyrrhiza inflata* Bat., *Glycyrrhiza glabra* L	Root, rhizome	Triterpenoid saponins, flavonoids, chalcones, coumarin etc.	Zhang and Ye (2009)
	*Scutellaria baicalensis* Georgi	Root	Baicalin, baicalein etc.	Ji et al. (2015)
	*Houttuynia cordata* Thunb	Whole herb, overground parts	Chlorogenic acid, scopolamine, quercetin, rutin, isoquercetin, vitexin etc.	Yang et al. (2014)
	*Terminalia chebula* Retz., *Terminalia chebula* Retz. var. tomentella Kurt	Ripe pod	Gallic acid, gallicin, corilagin, ellagic acid etc.	Chen et al. (2017)
Traditional Chinese formula	Ge-gen decoction (GGD)	*Puerariae lobatae radix*; *Ephedrae herba*; *Cinnamomi ramulus*; *Glycyrrhizae radix et rhizoma*; *Paeoniae radix alba*; *Zingiberis rhizoma recens*; *Jujubae fructus*	Puerarin, daidzein, paeoniflorin, cinnamic acid, glycyrrhizic acid, ephedrine, pseudoephedrine etc.	Song et al. (2007)
	San-wu-huang-qin decoction (SWHQD)	*Sophorae flavescentis radix*; *Scutellariae radix*; *Rehmanniae radix*	Verbascoside, baicalin, wogonoside, baicalein, matrine, sophocarpine, oxymatrine, oxysophorcarpine etc.	Ma et al. (2018)
	Gu-ben-fang-xiao decoction (GBFXD)	*Astragali radix*; *Codonopsis radix*; *Atractylodis macrocephalae rhizoma*; *Poria*; *Ostreae concha*; *Cicadae periostracum*; *Citri reticulatae pericarpium*; *Saposhnikoviae radix*; *Magnoliae flos; Schisandrae chinensis fructus*; *Glycyrrhizae radix et rhizoma*	——	
	Qing-fei-pai-du decoction (QFPDD)	*Ephedrae herba*; *Glycyrrhizae radix et rhizoma*; *Armeniacae semen amarum*; *Gypsum fibrosum*; *Cinnamomi ramulus*; *Alismatis rhizoma*; *Polyporus*; *Atractylodis macrocephalae rhizoma*; *Poria*; *Bupleuri radix*; *Scutellariae radix*; *Pinelliae rhizoma*; *Zingiberis rhizoma recens*; *Asteris radix et rhizoma*; *Farfarae flos*; *Belamcandae rhizoma*; *Asari radix et rhizoma*; *Dioscoreae rhizoma*; *Aurantii fructus immaturus*; *Citri reticulatae pericarpium*; *Pogostemonis herba*	Ephedrine, amygdalin, nobiletin, liquiritin, gallic acid, chlorogenic acid, saikosaponin A, glycyrrhizic acid etc.	Liu et al. (2021b)
	Ma-xing-shi-gan decoction (MXSGD)	*Ephedrae herba*; *Armeniacae semen amarum*; *Glycyrrhizae radix et rhizoma*; *Gypsum fibrosum*	Ephedrine, pseudoephedrine, amygdalin, glycyrrhizic acid etc.	Li et al. (2021)
	Ma-huang-xi-xin-fu-zi decoction (MXFD)	*Ephedrae herba*; *Aconiti lateralis radix praeparata*; *Asari radix et rhizoma*	Methylephedrine, aconine, songorine, fuziline, neoline, talatisamine, chasmanine, benzoylmesaconine, benzoylaconitine, deacetylhypaconitine etc.	Sun et al. (2016), Liang et al. (2020c)
Proprietary Chinese medicine	Lian-hua-qing-wen capsules (LHQWC)	*Forsythiae fructus*; *Ephedrae herba*; *Lonicerae japonicae flos*; *Isatidis radix*; *Menthae haplocalycis herba*; *Dryopteridis crassirhizomatis rhizoma carbonisatum*; *Rhodiolae crenulatae radix et rhizoma*; *Gypsum fibrosum*; *Pogostemonis herba*; *Rhei radix et rhizoma*; *Houttuyniae herba*; *Glycyrrhizae radix et rhizoma*; *Armeniacae semen amarum*	Salidroside, chlorogenic acid, forsythin E, cryptochlorogenic acid, amygdalin, swainonine, hyperoside, rutin, forsythin A, forsythin, rhein, glycyrrhizic acid etc.	Jia et al. (2015)
	Yin-hua-ping-gan granules (YHPGG)	*Puerariae lobatae radix*; *Lonicerae japonicae flos*; *Polygoni cuspidati rhizoma et radix*; *Ephedrae herba*; *Armeniacae semen amarum*; *Glycyrrhizae radix et rhizoma*	Ephedrine, pseudoephedrine, chlorogenic acid, amygdalin, puerarin, polygonin, glycyrrhizic acid, rheum emodin etc.	Du et al. (2018)
	Shu-feng-jie-du capsules (SFJDC)	*Polygoni cuspidati rhizoma et radix*; *Forsythiae fructus*; *Isatidis radix*; *Bupleuri radix*; *Patriniae herba*; *Verbenae herba*; *Phragmitis rhizoma*; *Glycyrrhizae radix et rhizoma*	Forsythin, forsythin E, rheum emodin, verbenalin etc.	Sun et al. (2016)
	Re-du-ning injection (RDNI)	*Artemisiae annuae herba*; *Lonicerae japonicae flos*; *Gardeniae fructus*	Iridoids, lignans, phenolic acids, flavonoids, caffeoylquinic acids, sesquiterpenes, coumarin etc.	Chen et al. (2020)
	Xue-bi-jing injection (XBJI)	*Carthami flos*; *Paeoniae radix rubra*; *Chuanxiong rhizoma*; *Salviae miltiorrhizae radix et rhizoma*; *Angelicae sinensis* *radix*	Hydroxysafflor yellow A, paeoniflorin, albiflorin, senkyunolide I, benzoylpaeoniflorin etc.	Sun et al. (2017b)
	Tan-re-qing injection (TRQI)	*Scutellariae radix*; *Fel Selenarcti*; *Cornu Naemorhedi*; *Lonicerae japonicae flos*; *Forsythiae fructus*	Rutin, baicalin, baicalein, chrysin-7-*O*-indole-glucoside, baicalein 7-*O*-β-d-glucopyranoside, wogonin, cynaroside, chlorogenic acid, caffeic acid, ursodesoxycholic acid, chenodeoxycholic acid etc.	Li et al. (2019)

**TABLE 2:** TCMs that target viruses directly.

TCM	Extract/components	Virus type	*In vitro*/vivo	Mechanism of action	IC_50_/CC_50_/SI/K_D_	References
*Scutellaria baicalensis* Georgi	Ethanolic extract	SARS-CoV-2	*In vitro*	1. Inhibits viral replication and entry of virus into cells	1. IC_50_ = 0.74 μg/ml	Liu et al. (2021a)
				2. Inhibits SARS-CoV-2 3CLpro	2. IC_50_ = 8.52 μg/ml	
*Scutellaria baicalensis* Georgi	Scutellarin, dihydromyricetin, quercetagetin, myricetin	SARS-CoV-2	*In vitro*	Inhibits SARS-CoV-2 3CLpro	IC_50_ = 1.2–5.8 μM	Liu et al. (2021a)
*Houttuynia cordata* Thunb	Aqueous extract	SARS-CoV	*In vivo* and vitro	1. Inhibits SARS-CoV 3CLpro		Lau et al. (2008)
				2. Inhibits RdRp		
*Terminalia chebula* Retz	Chebulagic acid, chebulinic acid	H1N1	*In vitro*	Inhibits viral replication and NA-mediated viral release	IC_50_ = 1.36 ± 0.36 μM	Li et al. (2020a)
					IC_50_ = 1.86 ± 0.98 μM	
					CC_50_ > 100 μM	
*Glycyrrhiza uralensis* Fisch	Aqueous extract	RSV	*In vitro*	Prevents viral attachment and internalization	IC_50_ = 74.8–70.7 μg/ml	Feng Yeh et al. (2013)
					CC_50_ = 2010.4–1945.3 μg/ml	
					SI = 26.9–27.5	
*Glycyrrhiza uralensis* Fisch	18β-Glycyrrhetinic acid	RSV	*In vitro*	Prevents viral attachment and internalization	IC_50_ = 4.3–4.5 μg/ml	Feng Yeh et al. (2013)
					CC_50_ = 71.5–76.3 μg/ml	
					SI = 15.0–17.7	
MXSGD		H1N1	*In vitro*	1. Inhibits viral RNA and protein synthesis	1. IC_50_ = 0.83 ± 0.41 mg/ml CC_50_ = 71.5 mg/ml	Hsieh et al. (2012)
				2. Prevents viral attachment	2. IC_50_ = 0.58 ± 0.07 mg/ml	
				3. Prevents viral entry by regulating the PI3K/AKT signaling pathway	3. IC_50_ = 0.47 ± 0.08 mg/ml	
GGD		RSV	*In vitro*	1. Inhibits viral attachment and internalization	IC_50_ = 45.6–160.8 μg/ml	Chang et al. (2012)
				2. Stimulates IFN secretion	CC_50_ > 3 mg/ml	
GGD		IAV	*In vitro*	Blocks virus-induced PI3K/AKT signaling pathway, causing retention of viral NP in the nucleus		Wu et al. (2011)
SWHQD		H1N1	*In vitro* and vivo	1. Inhibits viral HA, NA, NP, and M2 proteins	CC_50_ = 12.76 mg/ml	Ma et al. (2018)
				2. Reduces virus titers in mouse lung tissue		
LHQWC		SARS-CoV-2	*In vitro*	Inhibits viral replication	IC_50_ = 411.2 μg/ml	Runfeng et al. (2020)
					CC_50_ = 1,089–1,157 μg/ml	
LHQWC		IAV (H1N1, H3N2, H6N2, H9N2, H7N9), IBV	*In vitro*	1. Acts in the early stage of viral infection	IC50 = 0.2–2 mg/ml	Ding et al. (2017)
				2. Inhibits NF-κB pathway and impairs nuclear export of viral RNP.	SI = 1.59–15.85	
RDNI		SARS-CoV-2	*In vitro*		CC_50_ = 0.047 mg/ml	Jia et al. (2021)
					IC_50_ = 2.405 μg/ml	
YHPGG		H1N1	*In vitro*	1. Inhibits viral replication	1. IC_50_ = 100.9 ± 8.0 μg/ml, SI = 26.4	Du et al. (2018)
				2. Inhibits viral adhesion	2. IC_50_ = 230.6 ± 27.3 μg/ml, SI = 11.6	
Baicalin		SARS-CoV-2	*In vitro*	Inhibits SARS-CoV-2 3CLpro	IC_50_ = 34.71 μM	Jo et al. (2020)
Baicalein		SARS-CoV-2	*In vitro*	Inhibits viral replication by mPTP-dependent interference in mitochondrial oxidative phosphorylation	IC_50_ = 10 μM	Huang et al. (2020a)
Baicalein		SARS-CoV-2	*In vitro*	1. Inhibits viral replication and acts on viral post-entry stage	1. IC_50_ = 2.9 μM	Liu et al. (2021a), Song et al. (2021)
				2. Inhibits SARS-CoV-2 3CLpro	2. IC_50_ = 0.39 μM	
Baicalein		H5N1	*In vitro*	1. Inhibits viral replication	IC_50_ = 18.79 ± 1.17 μM	Sithisarn et al. (2013)
				2. Inhibits NP production	SI = 5.82	
Baicalin		RSV	*In vitro* and vivo	Blocks viral adhesion and replication, and decreases RSV titer in mouse lung tissue	IC50 = 19.9 ± 1.8 μM	Shi et al. (2016)
					CC_50_ = 370 ± 10 μM	
Rheum emodin		SARS-CoV HCoV-OC43	*In vitro*	Suppresses viral 3A protein to inhibit viral release	K_1/2_ = 20 μM	Schwarz et al. (2011)
Rheum emodin		SARS-CoV	*In vitro*	Blocks interaction of SARS-CoV spike protein with ACE2	K_1/2_ = 200 μM	Ho et al. (2007)
Forsythin		SARS-CoV-2 HCoV-229	*In vitro*	Inhibits viral replication	CC_50_ = 1,034–1959 μg/ml	Ma et al. (2020)
					IC_50_ = 63.90–64.53 μg/ml	
					SI = 16.02–30.66	
Quercetin		SARS-CoV-2	*In vitro*	1. Inhibits SARS-CoV-2 3CLpro	1. Binding affinity = −6.25 kcal/mol, K_i_ = 7 μM	Abian et al. (2020), Derosa et al. (2021)
				2. Inhibits SARS-CoV-2 PLpro	2. Binding affinity = −4.62 kcal/mol	
Glycyrrhizic acid		H3N2	*In vitro*	Interacts with cell membrane to reduce endocytic activity and virus uptake		Wolkerstorfer et al. (2009)
Glycyrrhizic acid		SARS-CoV-2	*In vitro*	Inhibits ACE2		Luo et al. (2020), Yu et al. (2021)

**TABLE 3:** Immunomodulatory TCMs.

TCM	Extract/composition	Virus type/symptom	*In vitro*/vivo	Mechanism of action	References
*Houttuynia cordata *Thunb	Polysaccharides	H1N1	*In vivo* and vitro	1. Reduces expression of chemokine CCL20 in lungs and regulates balance of Th17/Treg carrying CCR6^+^	Shi et al. (2020a)
				2. Inhibits Th17 cell differentiation by downregulating phospho-STAT3	
*Houttuynia cordata *Thunb	Polysaccharides	H1N1	*In vivo*	Increases content of SIgA and ZO-1 in intestine to regulate gut-lung axis	Zhu et al. (2018)
*Isatis indigotica* Fort	Erucic acid	H1N1	*In vivo* and vitro	Reduces CD8^+^ CTL recruitment and pro-apoptotic signaling and inactivates NF-κB and p38 MAPK signaling	Liang et al. (2020b)
*Isatis indigotica* Fort	4(3H)-Quinazolone	RSV	*In vitro*	Inhibits expression of RIG-I and interferon regulatory factor 3 to suppress the transcription of IFN-β	He et al. (2017)
*Isatis indigotica* Fort	Epigoitrin	H1N1	*In vivo*	Reduces protein expression of MFN2 to increase expression of MAVs, and increases the production of IFN-β and IFITM3	Luo et al. (2019)
MXSGD		H1N1	*In vivo*	Decreases expression of CCL5 and CXCL10 in lung tissue to increase the growth of beneficial bacteria and improve the lung microecological environment and immune microenvironment	Wang et al. (2020b), Wang et al. (2021b)
GGD		H1N1	*In vitro* and vivo	Decreases expression of TNF-α and improves Th1/Th2 immune balance	Geng et al. (2019)
QFPDD		HCoV-229E	*In vitro*	Increases expression of IFN and ISGs to inhibit viral replication and acts at the early stage of viral infection	Wang et al. (2021a)
		HCoV-OC43			
MXFD		H1N1	*In vivo*	Improves glucose metabolism, and regulates arachidonic acid metabolism and glycerophospholipid and sphingolipid metabolic pathways	Sun et al. (2017a), Li et al. (2017b)
GBFXD		Asthma	*In vivo*	1. Regulates Th17/Treg balance and suppresses M2 macrophage polarization	Liu et al. (2019), Liang et al. (2020a), Dong et al. (2020)
				2. Inhibits expression of BAFF and BAFF-related receptors to reduce B cell activation and IgE release	
GBFXD		RSV, asthma	*In vivo*	1. Regulates fatty acid metabolism by activating AMPK pathway	Xing et al. (2019), You et al. (2021)
				2. Regulates cholesterol transport and complement factor activation	
RDNI		Fever	*In vivo*	Regulates amino acid metabolism, lipid metabolism and energy metabolism	Gao et al. (2020)
XBJI		Sepsis, acute lung injury	*In vivo*	Regulates pathways of purine, glutathione, sphingomyelin, arachidonic acid, and phospholipid metabolism	Shi et al. (2018), Xu et al. (2018)
YHPGG		H1N1	*In vivo*	1. Downregulates mRNA and protein expression of Bax and caspase-3, and upregulates Bcl-2 expression in mouse lung tissue	Peng et al. (2015), Du et al. (2020)
				2. Increases levels of CD4^+^ and CD4+/CD8^+^ and reduces levels of CD8^+^ in whole blood	
SFJDC	Verbenalin, forsythin	Acute lung injury	*In vivo*	Regulates expression of the ERK pathway	Li et al. (2017a)
	rheum emodin				
SFJDC	Forsythin E, verbenalin	H1N1	*In vitro*	Acts on type I IFN and NF-κB/MAPK signaling pathways	Tao et al. (2020)
	rheum emodin				
Baicalin		H1N1	*In vivo* and vitro	Induces IFN-γ production in human CD4^+^ and CD8^+^ T cells and NK cells, and activates JAK/STAT-1 signaling pathway	Chu et al. (2015)
Baicalin		H1N1	*In vivo* and vitro	Downregulates miR-146a expression and produces IFN to inhibit viral replication	Li and Wang (2019)
		H3N2			
Baicalin		H1N1	*In vivo* and vitro	Triggers macrophage M1 polarization and IFN activation to inhibit viral replication	Geng et al. (2020)
		H1N1			
Baicalin		H1N1	*In vivo*	Downregulates key factors in the RLRs signaling pathway to inhibit viral replication, and decreases T1/T2 and T17/Treg ratios to balance host inflammatory response	Pang et al. (2018)
Baicalin		H3N2	*In vitro*	Suppresses expression of Atg5–Atg12 and LC3-II, and attenuates autophagy	Zhu et al. (2015)

**TABLE 4:** TCMs that control inflammatory factors.

TCM	Extract/composition	Virus type/symptom	*In vitro*/vivo	Mechanism of action	References
*Isatis indigotica* Fort	Polysaccharides	H1N1	*In vitro*	Inhibits TLR3 pathway expression to decrease IL-6, CXCL10, MIG, and CCL5 expression	Li et al. (2017c)
*Houttuynia cordata* Thunb	70% ethanolic extract	H1N1	*In vivo*	Decreases CCL2, IL-8, TNF-α, and MDA levels by inhibiting TLR pathway	Ling et al. (2020)
*Glycyrrhiza uralensis* Fisch	Ethanolic extract	H1N1	*In vitro*	Inhibits CCL5 secretion to reduce inflammation	Ko et al. (2006)
MXSGD		H1N1	*In vivo*	Regulates CCL2 protein expression	Zou et al. (2018)
MXFD		H1N1	*In vivo*	Suppresses IL-6, CCL2, and TNF-α expression, and increases IL-10 expression	Rong et al. (2016)
SWHQD		H1N1	*In vivo*	Decreases IL-6, TNF-α, IL-1β, and IFN-γ levels, and increases IL-4 level	Ma et al. (2021)
GBFXD		RSV, asthma	*In vivo*	1. Decreases ORMDL3, TGF-β, and IL-6 levels	(Huang et al., 2016; Lu et al., 2016)
				2. Increases CXCL1 and IFN-γ levels	
LHQWC		SARS-CoV-2	*In vitro*	Reduces TNF-α, IL-6, CCL2, and CXCL10 production	Runfeng et al. (2020)
LHQWC		IAV	*In vivo*	Suppresses NF-κB activation and downregulates IL-6, IL-8, TNF-α, CXCL10, and CCL2 gene expression	Ding et al. (2017)
LHQWC		IBV	*In vitro*	Inhibits excessive expression of CCL5, IL-6, IL-8, CXCL10, TNF-α, CCL2, MIP-1β, and IFN-λ	Yang et al. (2020)
TRQI		Airway inflammation	*In vivo*	Reduces TNF-α, IL-1β, IL-6, and IL-8 release, mitigates mucus hypersecretion, suppresses NF-κB p65, ERK1/2, JNK, and p38 MAPK phosphorylation, and inhibits p38 MAPK and NF-κB p65 expression	Liu et al. (2016)
RDNI		H1N1	*In vivo*	Downregulates ROS, IL-1β, IL-18, and NLRP3 expression, and the translation of caspase-1	Chen et al. (2020)
RDNI		Sepsis	*In vivo*	Inhibits TNF-α, IL-6, IL-10 and MIP-2 expression, and HMGB1-mediated activation of TLR4/NF-κB/MAPKs signaling pathways	Wang et al. (2021d)
XBJI	Safflor yellow A, hydroxysafflor yellow A, anhydrosafflor yellow B	Acute lung injury	*In vivo* and vitro	Reduces levels of MPO and MPO-DNA complex in serum, and phosphorylation of c-Raf, MAPKK, and ERK.	Wang et al. (2020c)
YHPGG		H1N1	*In vivo*	Inhibits the expression of TLR4, MyD88, TRAF6, and NF-κB p65 pathways to increase IL-2 and IFN-γ levels and decrease IL-4, IL-5, and TNF levels	Peng et al. (2016a), Peng et al. (2016b)
YHPGG		H1N1	*In vitro*	Upregulates IFN-β, MX-1, ISG-15, and ISG-56 levels, downregulates IL-6 and TNF-α levels and protein expression of phosphorylated TBK1, IRF3, ERK1/2, P38 MAPK, and NF-κB p65, and increases phosphorylated STAT1 levels	Du et al. (2018)
Forsythin		SARS-CoV-2	*In vitro*	Inhibits NF-κB pathway to reduce the mRNA expression of TNF-α, IL-6, IL-1β, CCL2, and CXCL10	Ma et al. (2020)
		HCoV-229E			
Forsythin		H1N1	*In vivo*	Decreases the virus titers, IL-6 levels, and HA expression	Qu et al. (2016)
Glycyrrhizic acid		H5N1	*In vitro*	Reduces NF-κB, JNK, and p38 activation to decrease CXCL10, IL-6, CCL2, and CCL5 expression	Michaelis et al. (2011)
Glycyrrhizic acid		SARS-CoV-2	*In vitro*	Decreases HMGB1 levels and attenuates IL-1β, IL-6, and IL-8 release	Gowda et al. (2021)

**TABLE 5:** Tissue-protecting TCMs.

TCM	Tissue	*In vitro*/*in vivo*	Mechanism of action	References
*Scutellaria baicalensis* Georgi	Lung	*In vivo*	Reduces infiltration of excessive inflammatory factors	Zhi et al. (2019)
SFJDC	Olfactory epithelium	*In vivo*	Protects against neuronal apoptosis and rescues impaired autophagy	Mei et al. (2020)
LHQWC	Lung	*In vivo*, *in vitro*	Inactivates NF-κB and reverses SOCS3 expression in inflammatory macrophages by regulating JNK/API pathway	Li et al. (2020b)
XBJI	Liver	*In vivo*	Decreases levels of ALT and AST in serum, downregulates AST expression, downregulates TNF-α, IL-6 expression, and upregulates IL-10 and SOCS1 expression	Li et al. (2016)
Baicalein	Lung	*In vivo*	Decreases serum levels of IL-1β and TNF-α	Song et al. (2021)
Quercetin	Kidney	*In vitro*	Blocks activation of signaling pathways related to inflammation and apoptosis	Gu et al. (2021)
Quercetin	Lung	*In vivo*	Prevents chronic obstructive pulmonary disease exacerbation and pulmonary disease progression	Farazuddin et al. (2018)

### Direct-Acting Antivirals

**FIGURE 2:**
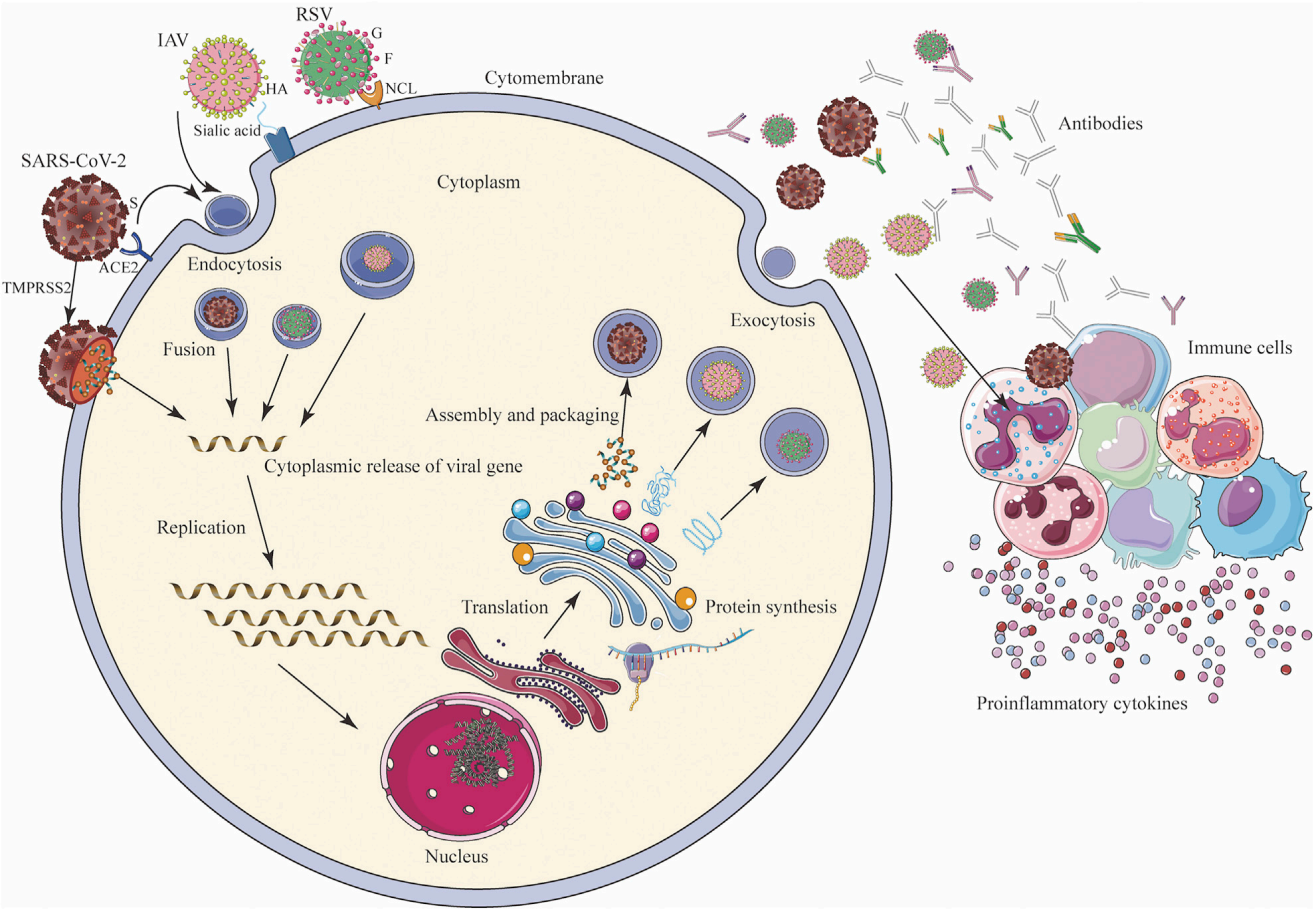
Mechanism of viral infection (SARS-CoV-2, IAV, and RSV) of host cells and host immune response. SARS-CoV-2 binds to ACE2, and IAV interacts with sialic acid through the HA on the surface and enters the host cells by endocytosis, and SARS-CoV-2 can directly enter cells under the action of TMPRSS2. G protein on the surface of RSV adheres to the cell membrane, and F protein binds to NCL and endocytosis into cells. After entering the cell, the virus releases its genome in the cytoplasm. Through transcription and translation in the nucleus, it is exported to the nuclear endoplasmic reticulum and ribosomes for the synthesis and assembly of viral proteins, and finally forms new progeny virus particles, which are exported to the outside of the cell in the form of exocytosis, and the host’s immune system While being activated, immune cells secrete a large number of antibodies and cytokines to fight the virus. ACE2: angiotensin converting enzyme 2; HA: hemagglutinin; G: glyco protein; F: fusion protein; NCL: nucleolin receptor; S: spike protein; TMPRSS2: transmembrane protease, serine 2.

Taking respiratory viruses, such as high-risk coronaviruses (SARS-CoV and SARS-CoV-2), IAV, and RSV, as examples, the direct inhibitory effects of TCMs on respiratory viruses include interference with viral adsorption and invasion, replication (e.g., transcription and translation, nuclear output, and assembly), packaging, and budding (Figure 2; Table 2).

#### Inhibition of Viral Adsorption and Invasion

The interaction between the viral surface protein and the host cell surface receptor is key for how the virus enters the cell; for example, the spike protein and angiotensin converting enzyme 2 (ACE2) receptor for SARS-CoV and SARS-CoV-2, hemagglutinin (HA) and the sialic acid protein for IAV, and the fusion protein and nucleolin receptor for RSV (Griffiths et al., 2020). Chinese herbal medicine aimed at the surface proteins or host receptors of these viruses can effectively “keep the enemy out of the country”. For example, RDNI acts on ACE2 to inhibit SARS-CoV-2 invasion, and effectively blocks viral replication in cells by inhibiting the main protease, resulting in a dual-target protective effect (Jia et al., 2021). The active component of LHQWC shared 189 common proteins with ACE2 co-expression proteins, which interact with each other (Zheng et al., 2020) and exert a multi-target synergistic effect that may prevent drug resistance caused by using a single ACE2 inhibitor (Runfeng et al., 2020; Yang et al., 2020; Chen et al., 2021a). LHQWC was the first drug approved to treat COVID-19 in China during the pandemic due to its clinical efficacy. Further studies showed that rheum emodin blocks the binding of the SARS-CoV spike protein to ACE2 and inhibits virus infection with a K_1/2_ value of about 200 μM(Ho et al., 2007). In addition, glycyrrhizic acid acts on the ACE2 receptor and prevents SARS-CoV-2 from entering cells (Luo et al., 2020; Yu et al., 2021).

In influenza viruses, MXSGD targets HA protein and regulates the phosphoinositide 3-kinase/protein kinase B (PI3K/AKT) signaling pathway to block viral entry, blocks H1N1 virus RNA replication and protein synthesis, and has a synergistic effect with oseltamivir (Hsieh et al., 2012). Glycyrrhizic acid acts on the cell membrane, reduces its endocytosis activity, inhibits the entry of IAV into cells, and thus reduces virus uptake (Wolkerstorfer et al., 2009).

In RSV invasion, GGD may inhibit RSV fusion protein, inhibit viral adsorption and invasion, and stimulate host mucosal cells to produce interferon (IFN)-β (Chang et al., 2012). *In vitro* studies have shown that aqueous licorice extract and glycyrrhetinic acid can inhibit RSV attachment and entry into host cells (Feng Yeh et al., 2013). Baicalin can block pre-infection by directly killing RSV(Shi et al., 2016).

#### Inhibition of Viral Replication

In SARS-CoV viral replication, 3C-Like protease (3CLpro), papain-like protease (PLpro), and RNA-dependent RNA polymerase (RdRp) are the key proteases (Hilgenfeld, 2014), and are promising drug targets (Anand et al., 2003). The aqueous extract of *Houttuynia cordata* Thunb. inhibits SARS-CoV 3CLpro and RdRp *in vitro* (Lau et al., 2008). The ethanolic extract of *Scutellaria baicalensis* Georgi inhibits viral replication by acting on 3CLpro (Liu et al., 2021a). Baicalein interferes with mitochondrial oxidative phosphorylation (Huang et al., 2020a) and inhibits SARS-CoV-2 3CLpro in a mitochondrial permeability transition pore (mPTP)-dependent manner *in vitro* (Liu et al., 2021a; Song et al., 2021). Baicalin and other active components in *Scutellaria baicalensis* Georgi, such as scutellarin, dihydromyricetin, quercetagetin, and myricetin, also selectively inhibit SARS-CoV-2 3CLpro (Jo et al., 2020; Liu et al., 2021a). In addition, forsythin inhibits the replication of coronaviruses, such as SARS-CoV-2, *in vitro* (Ma et al., 2020; Su et al., 2020). Molecular docking has shown that quercetin inhibits the 3CLpro and PLpro targets of SARS-CoV-2 (Abian et al., 2020; Derosa et al., 2021).

The IAV genome encodes 11 genes, including those for neuraminidase (NA), matrix protein 1 (M1), matrix protein 2 (M2), HA, and nucleoprotein (NP). Blocking the release, replication, and synthesis of proteins related to influenza virus ribonucleoprotein (RNP) is effective for anti-influenza therapy (Dou et al., 2018; Umeoguaju et al., 2021). SWHQD inhibits the HA, NA, NP, and M2 ion channels of the influenza H1N1 virus and blocks the proliferation and replication of virus particles (Ma et al., 2018). GGD inhibits the PI3K/AKT pathway induced by IAV, resulting in the retention of virus RNP in the nucleus, and thus interferes with viral replication (Wu et al., 2011). YHPGG has shown the best inhibitory effect on the replication stage of H1N1 influenza virus with a selectivity index (SI) of 26.4 (Du et al., 2018). LHQWC inhibits different strains of SARS-CoV-2, IAV, and IBV. In the early stage of virus infection, LHQWC inhibits the activity of nuclear factor (NF)-κB, weakening the nuclear output of virus RNP and progeny reproduction, and, combined with oseltamivir, improved symptoms in IBV-infected mice (Ding et al., 2017). Meta-analysis showed that LHQWC is superior to oseltamivir in improving the symptoms of IAV infection and is similar to oseltamivir in clearing the virus without serious adverse reactions (Zhao et al., 2014). In addition, baicalein inhibits the production of H5N1 influenza virus NP and inhibits viral replication (Sithisarn et al., 2013).

#### Inhibition of Virus Release

In coronaviruses, the 3A ion channel mediates the virus release (Schwarz et al., 2014). Rheum emodin inhibits the 3A ion channel in coronaviruses, such as SARS-CoV, and inhibits the release of progeny virions with a K_1/2_ value of about 20 μM (Schwarz et al., 2011).

In IAV, the NA, HA, and M2 proteins are exported to the plasma membrane and used with viral RNP to produce IAV virions. Under NA catalysis, the newly assembled viruses are then transmitted from the host cell (Battles and McLellan, 2019; Blockus et al., 2020; Umeoguaju et al., 2021). The release of influenza virions is closely related to NA protein. The active components of the aqueous extract of *Terminalia chebula Retz.*, chebulagic acid and chebulinic acid, inhibit the activity of viral NA protein, break the binding of virions to sialic acids on infected cells, block the virus release, and have a strong inhibitory effect on oseltamivir-resistant influenza strains (Li et al., 2020a).

Compared with small molecular inhibitors, TCMs have the advantage of containing multiple components and can have a synergistic effect on multiple targets. These multi-component, multi-target antiviral effects of TCMs support the basic theory of TCM.

### Indirect Immune Regulation Against Viral Diseases

Many TCMs control the development of viral diseases by regulating the balance of the immune system and maintaining the stability of the internal environment of the body, which is the embodiment of the TCM principle of holism (Table 3).

#### Regulation of IFN Secretion

IFN is a broad-spectrum antiviral glycoprotein, which acts as a trigger, regulator, and effector of the immune system to participate in many physiological responses in virus infection (Malmgaard, 2004), and it is the most important cytokine (Richard, 2021). QFPDD upregulates the expression of IFN and interferon-stimulated genes (ISGs), and acts on the early stage of SARS-CoV-2 virus infection (Wang et al., 2021a). Forsythin E, forsythin, verbenalin, and rheum emodin, which are the important components of SFJDC, improve the symptoms of mice infected with the H1N1 influenza virus by regulating type 1 IFN, the NF-κB/mitogen-activated protein kinase (MAPK) signaling pathway, and the extracellular signal-regulated kinase (ERK) pathway (Li et al., 2017a; Tao et al., 2020). Clinical studies have shown that SFJDC combined with umifenovir treatment improves the immunity of ordinary COVID-19 patients, inhibits pulmonary inflammation, and shortens the average antipyretic time (Chen et al., 2021b). *Isatis tinctoria L.* cooperatively regulates the expression of IFN-β by inhibiting the retinoic acid-inducible gene I (RIG-I) and melanoma differentiation-associated protein (MDA) 5 signaling pathways (Xu et al., 2019). The active components, tryptamine B, 4(3H)-quinazolone, and epigoitrin, activate the RIG-I signaling pathway, reduce the expression of mitochondrial fusion protein 2 (MFN2), and increase the expression of mitochondrial antiviral signal (MAVs), and thus promote IFN-β secretion (He et al., 2017; Luo et al., 2019). Baicalin downregulates the expression of miR-146a, which promotes IFN secretion and inhibits infection with H1N1 and H3N2 viruses (Li and Wang, 2019).

#### Regulation of Nonspecific and Humoral Immunity

After viral infection, cells recruit and activate macrophages, natural killer (NK) cells, and other immune cells by releasing cytokines, chemokines, and other signals to kill and eliminate infected cells. Once the regulation is out of balance, it causes an excessive immune response and tissue damage (Dai et al., 2020). GGD reduces the expression of the toll-like receptor (TLR) seven pathway signal and tumor necrosis factor (TNF)-α in H1N1 infected mice and improves the immune balance of T helper 1 (Th1) and T-helper 2 (Th2) cells (Geng et al., 2019). GBFXD inhibits the expression of B cell activating factor (BAFF) secreted by pulmonary macrophages and its related receptors, reduces the activation of B cells and the release of immunoglobulin E (IgE) (Liang et al., 2020a), inhibits the polarization activation of M2 macrophages, improves Th1/Th2 balance, reduces airway hyperresponsiveness and mucus secretion (Liu et al., 2019), regulates cholesterol transport, activates complement factors, and improves respiratory function and virus-induced asthma (Xing et al., 2019). YHPGG increases the level of CD4^+^T cells and the ratio of CD4^+^/CD8^+^ cells in peripheral blood of mice infected with H1N1, decreases the level of CD8+T cells (Peng et al., 2015), downregulates B-cell lymphoma 2 (Bcl-2)-associated X (Bax) and caspase-3 expression in mouse lung tissue, upregulates the expression of Bcl-2, and regulates apoptosis induced by virus (Du et al., 2020). Erucic acid in *Isatis tinctoria L.* reduces recruitment of CD8^+^ cytotoxic *T lymphocytes* (CTL), inhibits pro-apoptotic signals and NF-κB/MAPK signals, and reduces pulmonary inflammation (Liang et al., 2020b). Baicalin inhibits H1N1 infection by directly inducing human CD4^+^, CD8^+^T, and NK cells to produce IFN-γ and activating the JAK/STAT-1 signaling pathway (Chu et al., 2015) and by inducing macrophage M1 polarization and IFN activation (Geng et al., 2020). Furthermore, baicalin regulates key factors in the RIG-I-like receptors (RLRs) signaling pathway, inhibits H1N1 influenza viral replication, reduces the Th1/Th2 and T helper 17 (Th17)/regulatory T cells (Treg) ratios, and limits immunopathological damage (Pang et al., 2018). Baicalin also regulates the mTOR signaling pathway to inhibit the expression of the autophagy elongation complex (ATG5-Atg12) and lipidated LC3 (LC3-II), and inhibits autophagy induced by H3N2 influenza virus (Zhu et al., 2015).

#### Regulation of Intestinal Immunity

The lung and intestine originate from the same germinal layer in embryology and participate in mucus immunity (Barfod et al., 2013; Segal and Blaser, 2014; Wang and Tian, 2015). According to the theory of TCM, the lung and large intestine have an exterior-interior relationship. Intestinal disorders may affect the immune balance of lung tissue (Lee et al., 2021). When the intestinal barrier is damaged, pathogenic bacteria are exposed and transferred by M cells in the lymphoid follicular epithelium (Swank and Deitch, 1996), and infection of dendritic cells (DC) in gut-associated lymphoid tissue (GALT) activates T cell subsets in the mesenteric lymph node to produce regulatory cytokines (Wang et al., 2014). Intestinal mucosal immunity, which is central to the lung-intestinal axis, affects both the lung and the intestine (Zhu et al., 2018). MXSGD reduces the relative abundance of bacteria in the lung and intestine by reducing the levels of chemokines CC chemokine ligand (CCL) 5 and CXC motif chemokine ligand (CXCL) 10 in the lung tissue, promotes the growth of beneficial bacteria in the lung, improves the lung immune microecological environment, and protects the lungs from injury caused by the H1N1 influenza virus(Wang et al., 2020b; Wang et al., 2021b). Polysaccharides from *Houttuynia cordata* Thunb. promote specific migration of Th17CCR6^+^/TregCCR6^+^ cells from GALT to the lungs and regulate the Th17/Treg balance in IAV-infected mice (Shi et al., 2020a). In addition, these polysaccharides increase the levels of intestinal secretory immunoglobulin A (SIgA) and zonula occludens 1 (ZO-1), improve the intestinal physical barrier and immune barrier, inhibit the expression of TLR4 and p-NF-κB p65 in lung tissue, and reduce mortality in H1N1-infected mice (Zhu et al., 2018).

#### Regulation of Metabolism

Metabonomics is an important applied research method for the TCM principle of holism and uses a top-down strategy to understand physiological changes by analyzing the function of the organism based on the final effects in the metabolic network detected with modern techniques. The metabolic analysis of serum and feces by high-performance liquid chromatography time-of-flight mass spectrometry (TOF-MS) revealed that MXFD may exert an antiviral effect via mechanisms including improving energy metabolism and regulating arachidonic acid metabolism, glycerol phospholipid metabolism, the tricarboxylic acid cycle, tryptophan metabolism, and vitamin B6 metabolism (Sun et al., 2017a; Li et al., 2017b). Serum metabonomics showed that GBFXD activates the AMP-activated protein kinase (AMPK) pathway to regulate fatty acid metabolism and lipid metabolism and maintain the dynamic balance of lipids on the lung surface, and thus reduce asthma symptoms (You et al., 2021). Ultra-performance liquid chromatography to quadrupole (UPLC-Q)-TOF-MS analysis showed that RDNI regulates amino acid metabolism, lipid metabolism, and energy metabolism in febrile rats (Gao et al., 2020). Metabonomic analysis based on UHPLC-Q-Orbitrap high-resolution MS showed that XBJI reduces lung injury caused by sepsis by regulating energy metabolism, amino acid metabolism, fat metabolism, fatty acid metabolism, and hormone metabolism (Xu et al., 2018).

From the perspective of the principle of holism, TCMs regulate immune balance in many ways, such as IFN level, specific and non-specific immunity, intestinal immunity, and metabolism, and they reduce excessive immune reactions, suppress viral infection, improve pathological injury, and recover normal physiological function and the homeostasis of the internal environment.

### Control of Inflammatory Factors Induced by Viral Infection

Inflammation is part of the body’s immune system that helps to control viruses, but it can also cause pathological damage. Uncontrolled inflammation can trigger a cytokine storm, leading to cytokine release syndrome (Zuo et al., 2020), tissue damage to the heart, liver, and kidneys, respiratory and multiple organ failure, and even death (Schett et al., 2020; Huang et al., 2020b). Therefore, anti-inflammatory drugs are as important as antiviral drugs for critical patients (Table 4)(Ren et al., 2020; Zhang et al., 2020; Chikhale et al., 2021; Wang et al., 2021c).

#### Control of Inflammatory Factors to Prevent a Cytokine Storm

TCMs can synergistically regulate the release of cytokines and chemokines via multiple targets and pathways. MXFD inhibits the expression of interleukin (IL)-6, CCL2, and TNF-α in serum, increases the expression of IL-10 and reduces inflammatory reaction (Rong et al., 2016). SWHQD reduces the levels of IL-6, TNF-α, IL-1β, and IFN-γ, and increases the level of IL-4 in serum, bronchoalveolar lavage fluid (BALF), and lung tissue of H1N1 infected mice (Ma et al., 2021). MXSGD downregulates the expression of CCL2 protein in lung tissue (Zou et al., 2018). YHPGG increases IL-2 and TNF-γ levels and decreases IL-4, IL-5, and TNF levels in H1N1-infected mice by inhibiting the expression of the TLR4/myeloid differentiation primary response protein 88 (MyD88)/TNF receptor-associated factor 6 (TRAF6) signaling pathway and NF-κB p65 (Peng et al., 2016a; Peng et al., 2016b). In addition, YHPGG upregulates the levels of TNF-β and ISGs, such as *Mx-1*, *isg-15*, and *isg-56*, and regulates the protein expression of key effectors in the type I IFN and pattern recognition receptor signaling pathway (Du et al., 2018). LHQWC inhibits the cytokines TNF-α, IL-6, CCL2, and CXCL10 induced by SARS-CoV-2 *in vitro* (Runfeng et al., 2020); inhibits the activation of NF-κB induced by IAV and IBV; inhibits the gene expression of IL-6, IL-8, TNF-α, CXCL10, CCL2, and TNF-λ; and prevents severe inflammation (Ding et al., 2017; Yang et al., 2020). A randomized, double-blind, controlled clinical trial showed that the average antipyretic time of RDNI in the treatment of seasonal influenza was no longer than that of oseltamivir, and there were no serious adverse reactions (Liu et al., 2017). Treatment with ribavirin decreases the expression of reactive oxygen species (ROS) in lung tissue, downregulates IL-1β and IL-18 levels, and inhibits the activation of NLR family pyrin domain containing 3 (NLRP3) inflammatory bodies (Chen et al., 2020). Polysaccharides of *Isatis tinctoria L.* inhibit the expression of TLR3, and thus inhibit the secretion of CXCL10, IL-6, MIG, and CCL5(Li et al., 2017c). The 70% ethanolic extract of Houttuynia cordata Thunb. decreases the phosphorylation and nuclear translocation of TLR3/4/7 and NF-κB p65, and decreases the levels of CCL2, IL-8, TNF-α, and MDA (Ling et al., 2020). Glycyrrhizic acid reduces the activation of NF-κB, c-Jun N-terminal kinases (JNKs), and p38 and inhibits the expression of pro-inflammatory molecules, such as CXCL10, IL-6, CCL2, and CCL5, by inhibiting ROS formation induced by H5N1 (Michaelis et al., 2011). Forsythin reduces the production of proinflammatory cytokines TNF-α, IL-6, IL-1β, CCL2, and CXCL10 and alleviates cytokine storm caused by SARS-CoV-2 and human coronavirus (HCoV)-229E by regulating the NF-κB signaling pathway (Ma et al., 2020). In H1N1-infected mice, forsythin reduces the level of IL-6 and lung tissue injury (Qu et al., 2016).

#### Relief of Respiratory Symptoms Caused by Inflammation

Respiratory viruses cause acute asthma and airway inflammation via several mechanisms (Shi et al., 2020b). However, GBFXD increases the levels of CXCL1 and IFN-γ in the lungs and reduces the airway inflammation caused by RSV-ovalbumin (Lu et al., 2016). GBFXD may also prevent chronic asthma by reducing the levels of transforming growth factor (TGF)-β and IL-6, reducing the deposition of collagen in the airway, inhibiting the production of airway mucus, and downregulating the expression of orosomucoid like 3 (ORMDL3) (Huang et al., 2016). TRQI reduces the release of TNF-α, IL-1β, IL-6, and IL-8 in mouse lung tissue, reduces the entry of cytokines into BALF, reduces mucus secretion, regulates the NF-κB/MAPK signaling pathway, and alleviates respiratory tract inflammation (Liu et al., 2016). CCL5 plays an important role in activating and recruiting leukocytes to the inflammatory site. The ethanolic extract of Glycyrrhiza uralensis Fish.; Glycyrrhiza inflata Bat.; Glycyrrhiza glabra L. significantly inhibits the secretion of CCL5 by human bronchial epithelial cells induced by the H1N1 influenza virus (Ko et al., 2006).

#### Relief of Severe Inflammation

Severe COVID-19 patients exhibit symptoms including dyspnea, acute respiratory distress syndrome, and sepsis. At this stage, the mortality rate of patients is about 15% (Godeau et al., 2021). XBJI is the only proprietary Chinese medicine approved in China for treating sepsis, and it significantly shortens the improvement time for major clinical symptoms and hospital stays (Luo et al., 2021). The anti-inflammatory effect of XBJI arises from the regulation of the NF-κB signaling pathway (Zhou et al., 2021). Safflor yellow A, hydroxysafflor yellow A, and anhydrosafflor yellow B, the three main components of XBJI, inhibit increases in the levels of inflammatory factors in mouse BALF, reduce the level of plasma myeloperoxidase (MPO)-DNA complex, and decrease the phosphorylation of RAF proto-oncogene serine/threonine-protein kinase (c-RAF), mitogen-activated protein kinase kinase (MAPKK), and ERK in mouse lung tissue (Wang et al., 2020c). High mobility group protein B1 (HMGB1) is an important late inflammatory factor and an endogenous danger signal in the pathological process of sepsis (Andersson and Tracey, 2003). Luteolin, the active component of RDNI, inhibits the activation of the TLR4/NF-κB/MAPK signaling pathway mediated by HMGB1 (Wang et al., 2021d). Glycyrrhizic acid also inhibits the increase in HMGB1 after SARS-CoV-2 infection, reduces the levels of proinflammatory cytokines IL-1 β, IL-6, and IL-8, and alleviates severe inflammation (Gowda et al., 2021).

The infiltration of inflammatory factors induced by viruses can cause a variety of pathological damage in the focus tissue. TCMs can not only control cytokines and chemokines in many ways to prevent a cytokine storm, but can also protect critical patients, which demonstrates that TCMs can maintain the homeostasis of the internal environment of the body owing to their multiple components and targets.

### Tissue Protection

Respiratory viruses first invade the patient’s lungs, causing varying degrees of lung injury, and the viral infection may become systemic (Synowiec et al., 2021). Therapeutic drugs may also increase the load on the liver, kidneys, and other tissues, resulting in multi-tissue injury. TCMs can protect lung tissue and improve multiple organ function by inhibiting excessive apoptosis, inflammation, and immune reaction (Table 5).

LHQWC regulates the JNK/activator protein one signaling pathway, reduces the activity of NF-κB in macrophages, reverses the expression of suppressor of cytokine signaling (SOCS) three and the abnormal expression of TNF-related apoptosis-inducing ligand, protects cells from apoptosis, and alleviates acute lung injury in mice (Li et al., 2020b). The ethanolic extract of *Scutellaria baicalensis* Georgi reduces IL-6, TNF-α, and CCL2 levels in the lung tissue of H1N1-infected mice, increases IL-10 and IFN-γ production, and protects lung tissue, which is superior to the effect of active component baicalein alone (Zhi et al., 2019). In addition, baicalein inhibits the infiltration of inflammatory cells in lung tissue after RSV infection, decreases the serum levels of IL-1β and TNF-α, and improves respiratory function in acute lung injury in mice (Song et al., 2021). Quercetin reduces the inflammatory reaction and pathological deterioration of lung tissue in mice with chronic obstructive pulmonary disease induced by rhinovirus (Farazuddin et al., 2018).

In addition to protecting lung tissue, XBJI reduces the levels of serum alanine aminotransferase (ALT) and aspartate aminotransferase (AST), downregulates the expression of TNF-α and IL-6, upregulates the expression of IL-10 and SOCS1, and reduces liver injury caused by inflammation in rats (Li et al., 2016). Quercetin regulates apoptosis-related signaling pathways, blocks the inflammatory response, and protects against SARS-COV-2-induced acute kidney injury (Gu et al., 2021). Dysosmia is a common symptom in COVID-19 patients (Tong et al., 2020). SFJDC reduces the levels of IgE, TNF-α, and IL-1β in peripheral blood, lung tissue, and olfactory epithelial (OE) tissue of rats, prevents nerve cell apoptosis, rescues autophagy of damaged cells in lung and OE tissue, and protects OE neurons and lung tissue (Mei et al., 2020).

TCM regards the body as an organic whole, and thus not only regulates immune and inflammatory responses, but also prevents and protects from various pathological tissue injuries, resulting in mutual balance among various physiological functions. This overall stability and harmony are fundamental to disease prevention and health maintenance.

## Development Prospects of Antiviral TCMs

TCMs have become a main focus of antiviral research because of their advantages, including reliable clinical efficacy, few side effects, and low drug resistance, which arise from the principles of holism and syndrome differentiation and treatment. Therefore, to study the antiviral properties of TCMs and develop new TCMs, we must combine the basic theory of TCM with the latest research in Western medicine and modern biological science. In emergencies, Western medicine emphasizes using compounds that target a single receptor to relieve symptoms quickly at the disease site, which is effective, but not always sufficient to restore the functional balance of the body, making adverse reactions particularly obvious. For example, during the SARS epidemic in 2003, high-dose glucocorticoid treatment caused serious side effects in critical patients, including immunosuppression, delayed virus clearance, and bone destruction (Choudhry et al., 2020). Holistic treatment with TCMs, which have multiple components and targets, focuses on the interactions and relationships among the body, viruses, and drugs, and has considerable advantages in adaptability and effectiveness in the treatment of complex human diseases that cause immune imbalance, especially during an outbreak of an unknown new virus. This holistic philosophy is also being used in the emerging field of network pharmacology, and is recognized in modern research methods, such as network biology and metabonomics.

The greatest advantage of TCM is the coordination of multiple components and targets. Because of the many components, TCM does not rely on a single antiviral mechanism of action, but harnesses the coexistence and interaction of multiple mechanisms. However, there is still insufficient research on the chemical component analysis and mechanism of action of TCMs, which restricts the development of TCM, and is also an obvious short board in the modern medical system. (Li et al., 2018). And research on the safety of TCMs in the treatment of viral diseases are not sufficient. “Safe” of TCMs is not the same as “natural”. Some TCMs have endogenous toxicity to organs such as liver and kidney, and also exogenous toxicity in the process of cultivation, processing, storage and distribution (Li et al., 2020c). Therefore, the further development of TCMs in the world must determine its side effects. Under the guidance of TCM theory, research on the mechanism of TCMs should examine the relationship of infection and immune response between virus and host directly, revealing the dynamic relationships between viral load, cytokines, and immune response. This information may reveal new insights that are difficult to discover via traditional biology, explain the mechanism of prevention and treatment of viruses with herbs using the technology and language of modern life sciences, and promote the deep integration of TCM and modern biotechnology.
